# Downregulation of Squalene Synthase Broadly Impacts Isoprenoid Biosynthesis in Guayule

**DOI:** 10.3390/metabo12040303

**Published:** 2022-03-29

**Authors:** Dante Placido, Niu Dong, Bashar Amer, Chen Dong, Grisel Ponciano, Talwinder Kahlon, Maureen Whalen, Edward E. K. Baidoo, Colleen McMahan

**Affiliations:** 1 Western Regional Research Center, Agricultural Research Service, United States Department of Agriculture, Albany, CA 94710, USA; dante.placido@gmail.com (D.P.); niu.dong@usda.gov (N.D.); chen.dong@usda.gov (C.D.); grisel.ponciano@usda.gov (G.P.); talwinder.kahlon@usda.gov (T.K.); whalen.maureen@gmail.com (M.W.); 2 Joint Bioenergy Institute, Emeryville, CA 94608, USA; bamer@lbl.gov (B.A.); eebaidoo@lbl.gov (E.E.K.B.); 3 Biological Systems and Engineering Division, Lawrence Berkeley National Laboratory, Berkeley, CA 94720, USA

**Keywords:** *Parthenum argentatum*, guayule, squalene synthase, squalene, natural rubber

## Abstract

Production of natural rubber by *Parthenium argentaum* (guayule) requires increased yield for economic sustainability. An RNAi gene silencing strategy was used to engineer isoprenoid biosynthesis by downregulation of squalene synthase (SQS), such that the pool of farnesyl diphosphate (FPP) substrate might instead be available to initiate natural rubber synthesis. Downregulation of SQS resulted in significantly reduced squalene and slightly increased rubber, but not in the same tissues nor to the same extent, partially due to an apparent negative feedback regulatory mechanism that downregulated mevalonate pathway isoprenoid production, presumably associated with excess geranyl pyrophosphate levels. A detailed metabolomics analysis of isoprenoid production in guayule revealed significant differences in metabolism in different tissues, including in active mevalonate and methylerythritol phosphate pathways in stem tissue, where rubber and squalene accumulate. New insights and strategies for engineering isoprenoid production in guayule were identified.

## 1. Introduction

Guayule (*Parthenium argentatum* Gray), a perennial shrub indigenous to the Chihuahuan desert of Mexico and southwestern Texas, is one of 2000+ natural rubber-producing plant species [1,2] and is under development as a drought-tolerant industrial crop for the western United States. Natural rubber, a plant secondary metabolite, is a strategic and irreplaceable material vital to modern transportation, medicine, and defense (Critical Agricultural Materials Act 7, Public Law 107–293, U.S.C 178. 2019). In guayule, natural rubber is biosynthesized in stembark tissues [3], especially in response to abiotic and biotic stresses [4] such as cold, non-freezing temperatures [5,6,7]. Natural rubber biosynthesis requires the 15-carbon initiator, farnesyl pyrophosphate (FPP), and the 5-carbon monomer, isopentenyl pyrophosphate (IPP), both derived from the isoprenoid pathway. Plants are major sources of isoprenoids and produce a myriad of them through the cystosolic mevalonic acid (MVA) and chloroplastic methylerythritol phosphate (MEP) pathways [8,9,10].

Attempts to increase rubber yield in plants have included overexpression of farnesyl pyrophosphate synthase (*FPPS*) [11] and other genes for isoprenoid pathway enzymes [12,13,14]. Remarkably, overexpression of isoprenoid production genes in both rubber-producing [13] and non-rubber producing [15] plants led to very high production of the C-30 isoprenoid squalene, which accumulated in subcellular microbodies. Squalene is produced by the condensation of two molecules of FPP in a reaction catalyzed by squalene synthase (SQS, EC 2.5.1.21); consequently, biosynthesis of natural rubber and squalene share a common isoprenoid pathway substrate at the branching point between triterpene/steroid and polyisoprene biosynthesis [8].

Squalene synthase (SQS) is a structurally conserved enzyme found ubiquitously in nature [16,17]; it synthesizes precursors for critical isoprenoids [16]. SQS is considered an essential control point for the management of carbon flux in, for example, forming cholesterol in mammals and sterols and triterpenes in plants, depending on the cellular environment [8,9]. In plants, phytosterols are vital in plant growth and development [18,19], and triterpene compounds play an essential role in biotic responses [20]; thus, genetic manipulation of *SQS* has been studied in plants with considerable interest.

Examples of *SQS* engineering in plants include the manipulation of triterpene [20] and phytosterol production [19,21]. Overexpression of *SQS* from *Panax ginseng* (*PgSS1*) [22,23], *Bupleurum falcatum* (*BfSS1*) [24], *Arabidopsis thaliana* (*AtSS1*) [25], and *Glycine max* (*GmSQS1*) [26] increased phytosterol and triterpene biosynthesis, establishing *SQS*’s role in metabolic regulation. Suppression of *SQS* in *Artemisia annua* upregulated artemisinin biosynthetic genes, increasing the content of the antimalarial compound artemisinin [27]. Silencing SQS in *Withnoldes somifera*, an important medicinal plant in India, led to reduced squalene and withanolide content [28].

Physiologically and phenotypically, SQS-silenced plants showed decreased sterol content, white leaf pigmentation, and misshaped leaves in apple (*Malus* x *domestica)* [29]. In rice (*Oryza sativa*), RNAi-mediated transformation of maize squalene synthase impacted root morphology and reduced stomatal conductance, resulting in improved drought tolerance [30].

While considerable private and public investment over the last decade has verified guayule’s potential as an important industrial crop, increased natural rubber (NR) yield remains the primary goal of guayule improvement. In this report, we genetically downregulated squalene synthase expression in guayule by RNAi technology, such that the pool of FPP might be available to enhance natural rubber synthesis (Figure S1).

## 2. Results

**Figure 1:**
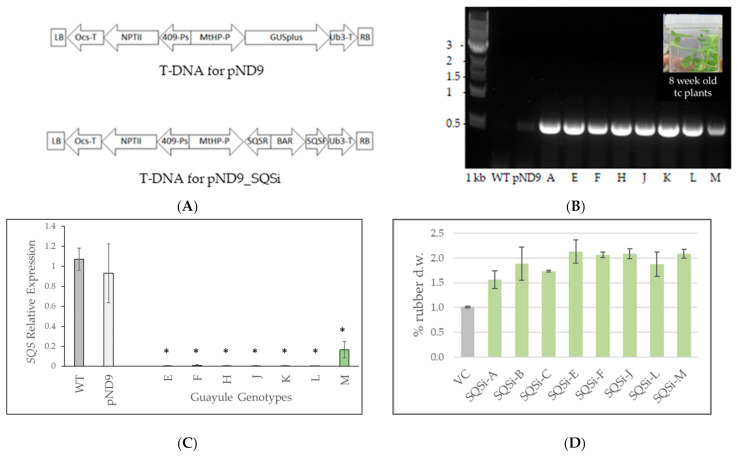
(**A**) T-DNA region of transformation constructs. pND9, β-glucuronidase plus (*GUSPlus*) reporter gene driven by *Medicago truncatula* promoter (MtHP-P) with ubiquitin 3 terminator (Ub3-T) and a shortened potato polyubiquitin promoter (409-Ps) controlling the expression of the neomycin phosphotransferase II (*nptII*) gene for kanamycin resistance with octopine synthase terminator (Ocs-T). pND9_SQSi, the *GUSPlus* gene in pND9, is replaced with a hairpin RNAi construct built with two inverted repeats of the partial *P. argentatum SQS* gene (SQSR and SQSF); a bialaphos resistance (*BAR*) gene was inserted between them. LB, left border, RB, right border. (**B**) Confirmation of transgenic plants. Guayule transgenic plants were confirmed with PCR using genomic DNA as a template. Primers for the BAR gene were used to confirm the guayule plants harboring pND9_*SQS* T-DNA. The expected product size is 551 bp in the downregulated lines. (**C**) Quantitative-RT-PCR (qPCR) of stembark tissues. Expression level of 170 bp PCR product in the *SQS* coding sequence compared with WT. The *18S* gene (195 bp) was used as an internal control. The asterisks (*) indicate a significant difference in comparison with WT at *p* ≤ 0.05. (**D**) Rubber content in tissue-cultured plants, as determined by ASE microassay. WT = G7-11 wildtype; VC = vector control pND9; SQSi-A, -E, -F, -H, -J, -K, -L, and -M represent independent gene downregulation events.

RNA interference was used to downregulate *SQS* in guayule line G7-11/AZ-2. This approach is a sequence-specific gene downregulation mediated by introducing double-stranded RNA into the cell, resulting in the degradation of the specific mRNA and function loss for the target gene [31,32]. To confirm positive *SQS*-transformed plants, the plasmid construct segment of the inserted bialaphos (BAR) gene sequence (Figure 1A) was amplified by polymerase chain reaction (PCR) from leaves of 8-week-old tissue cultured plants. Eight lines were deemed positively transformed (Figure 1B) and labeled SQSi. Quantitative reverse transcription Polymerase Chain Reaction (qPCR) analysis verified that the *SQS* gene was suppressed in the SQSi lines but not in the wildtype (WT) and vector control (VC) plants (Figure 1C). Phenotype screening of tissue-culture-grown plants showed, on average, 52% higher natural rubber content (Figure 1D) and 10% lower resin (squalene-containing fraction) content (not shown) for SQSi plants compared to the control. Three biological SQSi lines, J, L, and M, were selected for greenhouse/growth chamber phenotype characterization based on consistent results in replicated experiments.

When grown in soil, at 5 months, plant architectural features of *SQSi* lines did not show remarkable differences compared to WT in, for example, biomass, size, or SPAD (chlorophyll) readings (Figure S2), in contrast to reports in apple [29] and rice [30]. Surprisingly, significant differences were found between the WT control and the VC plants for number of branches and number of flowering buds.

**Figure 2:**
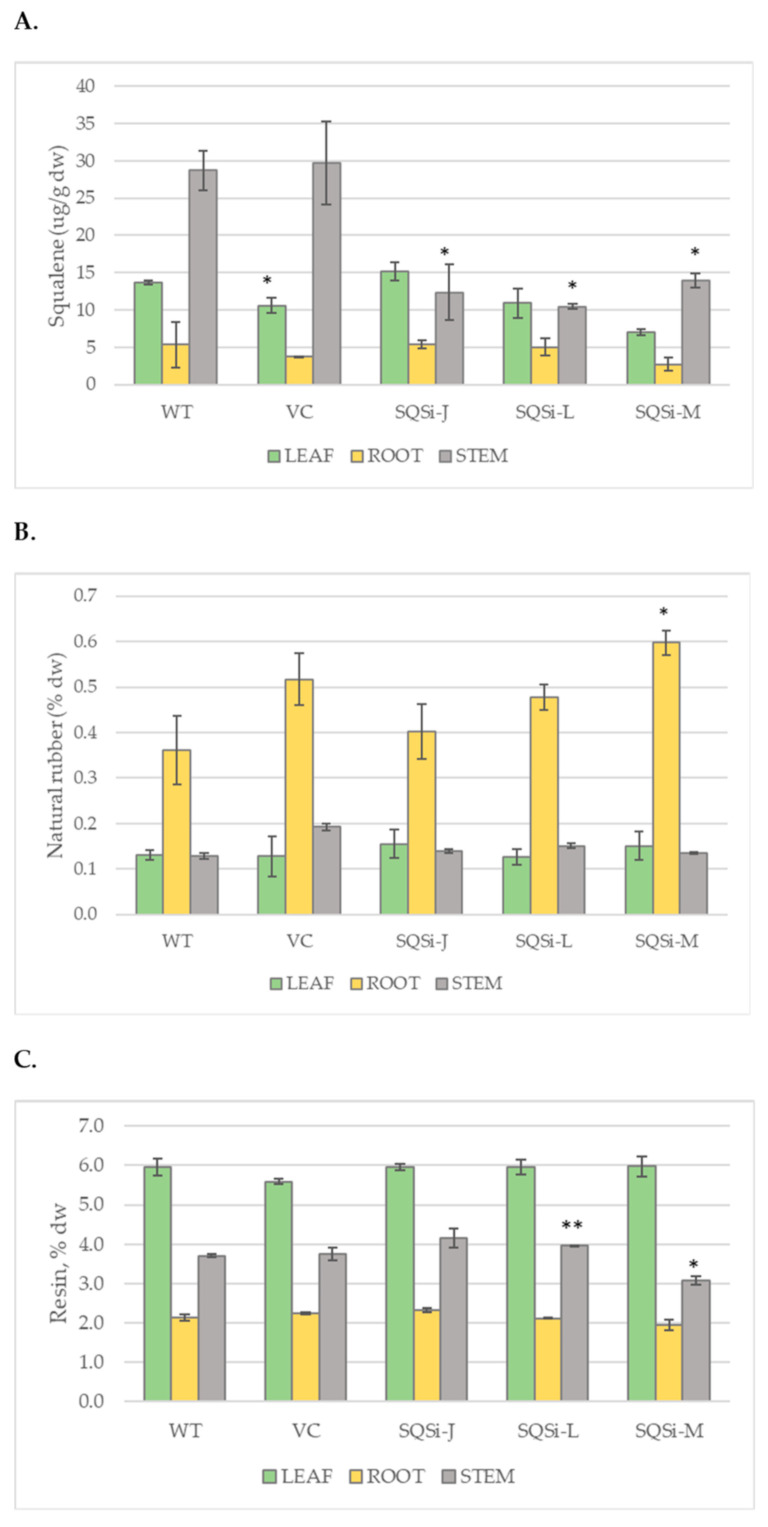
Secondary metabolites in guayule. Concentration of targeted metabolites in 5-month-old guayule plant tissues: WT = wildtype, VC = vector control, squalene synthase downregulated lines *SQSi*-J, -L, -M represent 3 independent transformation events. (**A**) Squalene (ug/g dw), determined from purified acetone extracted tissue samples using gas chromatography-mass spectrometry (GC-MS). (**B**) Natural rubber (% dw), and (**C**) resin (% dw), quantified by accelerated solvent extraction. Average value of 3 biological replicates; error bars are +/− standard error; symbol * denotes significantly different from WT at *p* < 0.05; ** at *p* < 0.005.

Guayule sequesters carbon dioxide and converts it to metabolites, including natural rubber, squalene, and ‘resin’, a mixture of terpenes, fatty acids, low molecular weight rubber, and other chemicals co-extracted during rubber biorefining processes. In our investigation, squalene content varied by tissue type and genotype for plants grown in greenhouse/growth chamber conditions. Analysis of control plants by gas chromatography-mass spectrometry (GC-MS) found the highest level of squalene in stem tissues (up to 30+ μg per g dry weight tissue), followed by leaf, then root (Figure 2A). All *SQSi* lines analyzed had less squalene in stem tissues, ~50–60% reduction compared with the WT (*p* < 0.05; Figure 2A). No significant differences were found in squalene content for leaf and roots tissues in *SQSi* lines compared with controls.

The natural rubber concentration for growth chamber-grown plants, at less than 1% dry weight (Figure 2B), was significantly lower than would be expected in field plants. The highest rubber concentration was found in root tissue, as had been previously reported in young [33] and mature [34] guayule. In root tissue, natural rubber production was enriched by up to 65% in *SQSi* lines (significantly in *SQSi*-M) compared to the WT but not compared to the VC line (Figure 2B). Less rubber was found in stem and leaf tissues; small increases in NR production in the *SQSi* lines’ stem tissue compared to the WT were not statistically significant and surprisingly lower than the VC. Resin concentration (up to 6% dw) was highest in leaf tissue, as expected (Figure 2C), and did not vary significantly in leaf or root tissues. Differences in resin content of stem tissue between *SQSi* lines and the WT control were noted but inconsistent (mixed higher and lower).

So, quite interestingly, downregulation of *SQS* resulted in significantly reduced squalene in all lines and enhanced rubber concentrations in some but not in the same tissues nor to the same extent. To gain insight into the pathway flux, concentrations of 13 additional precursor and intermediate compounds involved in natural rubber and squalene biosynthesis were quantified by liquid chromatography-mass spectrometry (LC-MS) (Table S1).

**Figure 3:**
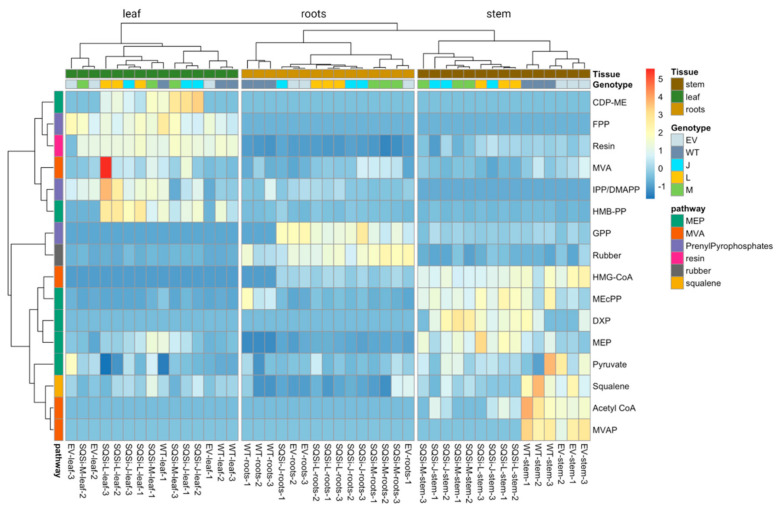
Multivariate analysis visualization of precursor and intermediate compounds involved in natural rubber and squalene biosynthesis across all control and *SQSi* lines and tissues. Individual samples with different genotype and tissue type appear on the vertical axis, and the pathway of each identified measured compound is represented in the horizontal axis. Relative abundance of targeted metabolites is represented by color; orange indicates high relative abundance, while blue indicates low abundance. Pearson’s correlation-based hierarchical cluster analysis indicates a strong separation across different tissue types (column-wise), while no clear clustering pattern was observed in terms of pathways (row-wise). The heatmap scale ranges from −1 to 5 (data pre-treated with autoscaling).

Multivariate analysis of the LC-MS results across both control and transgenic lines (Figure 3) illustrates distribution patterns in stem, leaf, and root tissue. The resulting heat map reveals the primary level of separation is by tissue type, dominating over genotype. Moreover, in stem tissue, WT and VC lines are grouped perfectly away from transgenic lines, suggesting the *SQSi* genetic modification has a more profound effect on metabolism in young stem tissue, where squalene is most concentrated in guayule. Levels of metabolites were dramatically different, especially between leaf tissue and stem tissue, as shown by the high abundance of orange blocks in the left upper corner and right lower corner of Figure 3. However, these blocks could not be assigned to specific pathways since MEP (chloroplastidic) and MVA (cytosolic) pathways did not show patterns of clustering (as shown by the left axis pathways’ color block distribution), suggesting a mixed and more complex underlying regulation between these two metabolic pathways. Further, correlation analysis (Figure S3) revealed strong and significant positive correlations between rubber and GPP (r = 0.69, *p* < 0.05), and resin and FPP (r = 0.77, *p* < 0.05). Squalene was positively correlated with both acetyl CoA and mevalonate-5-phosphate (MVAP).

**Figure 4:**
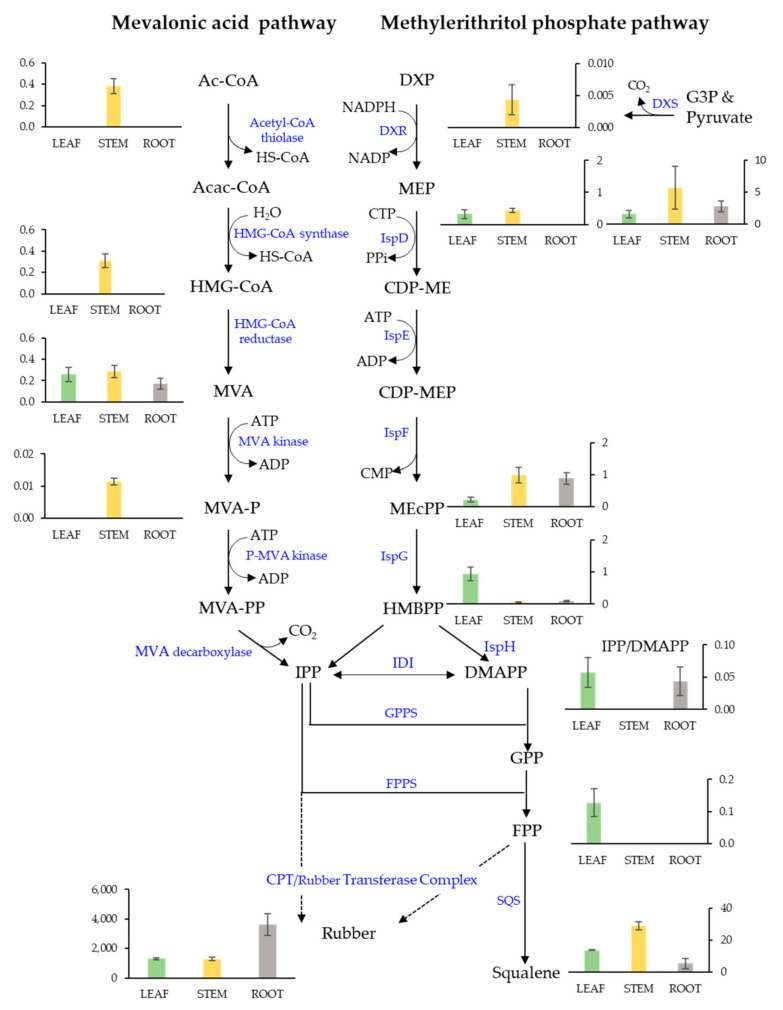
Isoprenoid metabolism in WT guayule. Concentrations (μg/g dry weight) of metabolites in 5-month-old guayule plant tissues including: (1) the cytosolic mevalonic acid Pathway intermediates: acetyl coenzyme A (AC-CoA); acetoacetyl coenzyme A (Acac-CoA); hydroxymethylglutaryl coenzyme A (HMG-CoA); mevalonate (MVA); mevalonate-5-phosphate (MVA-P); and mevalonate diphosphate (MVA-PP). (2) The methylerithritol phosphate pathway (chloroplast) intermediates: pyruvate and glyceraldehyde-3-phosphate (G3P); 1-deoxylulose-5-phosphate (DXP); methylerythritol-4-phosphate (MEP); 4-(cytidine-5′diphospho)-methylerythritol (CDP-ME); methylerythritol-2,4-cyclodiphosphate (CDP-MEP), 2-C-methyl-d-erythritol 2,4-cyclodiphosphate (MEcPP); and 4-hydroxy-3-methylbut-2-enyl diphosphate (HMBPP). Also shown, common products of both pathways: isopentenyl pyrophosphate (IPP)/dimethylallyl diphosphate (DMAPP), geranyl diphosphate (GPP), farnesyl diphosphate (FPP), squalene, and natural rubber. Error bars represent +/− 1 standard error.

Next, a more detailed analysis of the LC-MS data, the first of its kind for guayule, provided new insight into the isoprenoid pathway flux in guayule. The results for WT plants (Figure 4) clearly illustrate higher concentrations of mevalonate pathway metabolites, especially acetyl co-A, HMG-coA, and mevalonate, in WT stem tissues compared with leaf and root tissues. MEP pathway intermediates, including the precursor pyruvate, were also more abundant in stem tissue (except 4-hydroxy-3-methylbut-2-enyl diphosphate (HMBPP)). We note that for guayule, stem tissue, where rubber biosynthesis occurs, includes soft bark parenchyma tissue rich in chloroplasts. Leaf tissue presented the highest concentrations of HMBPP, isopenentyl pyrophosphate (IPP)/di-methylallyl diphosphate (DMAPP), and FPP. In fact, FPP, the primary substrate for squalene synthesis and the natural rubber initiator molecule, was only detected in leaf and not in stem or root tissue. Note that geranyl geranyl diphosphate (GGPP) was not detected in any tissues, and GPP was found mainly in root tissue (Table S1). In addition, mevalonate phosphate and pyrophosphate were at trace levels or not detected.

In comparing results across genotypes, we note that in the quantification of plant metabolites, there were several cases where the VC concentrations deviated significantly from those for the WT control (Table S1). Differences were, at times, systematic across tissue types, i.e., pyruvate concentrations in VC plants were higher than the WT in all 3 tissues; MEP and HMB-PP levels for VC were lower than the WT, also for all three tissues. While it is possible that gene insertion may have produced unintended effects, the specific reason for these differences is not known. Our analysis, therefore, primarily considered comparisons between *SQSi* and WT plants.

**Figure 5:**
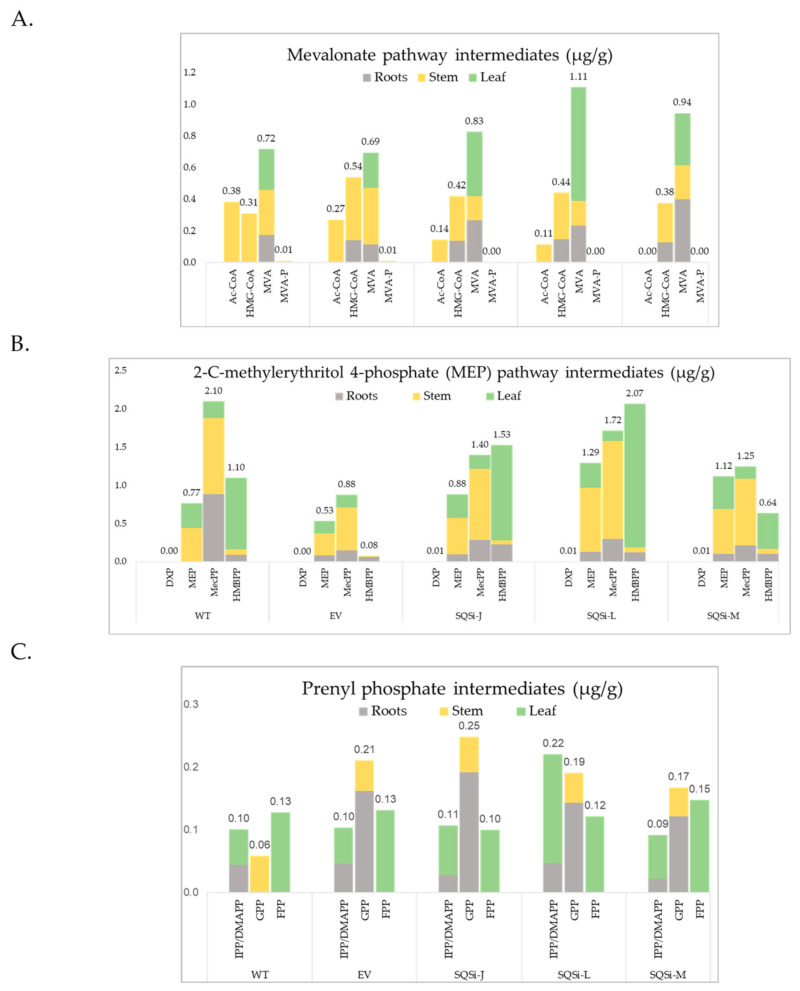
Effect of SQS downregulation on isoprenoid intermediates. Concentrations (ug/g dry weight) of metabolites in 5-month-old guayule plant tissues including: (**A**) the cytosolic mevalonic acid pathway intermediates: acetyl coenzyme A (AC-CoA); acetoacetyl coenzyme A (Acac-CoA); hydroxymethylglutaryl coenzyme A (HMG-CoA); mevalonate (MVA); mevalonate-5-phosphate (MVA-P); and mevalonate diphosphate (MVA-PP). (**B**) The methylerithritol phosphate pathway (chloroplast) intermediates: pyruvate and glyceraldehyde-3-phosphate (G3P); 1-deoxylulose-5-phosphate (DXP); methylerythritol-4-phosphate (MEP); 4-(cytidine-5′diphospho)-methylerythritol (CDP-ME); methylerythritol-2,4-cyclodiphosphate (CDP-MEP), 2-C-methyl-d-erythritol 2,4-cyclodiphosphate (MEcPP); and 4-hydroxy-3-methylbut-2-enyl diphosphate (HMBPP). (**C**) Also shown, common products of both pathways: isopentenyl pyrophosphate (IPP)/dimethylallyl diphosphate (DMAPP), geranyl diphosphate (GPP), farnesyl diphosphate (FPP), squalene, and natural rubber. Values represent the average of 3 biological reps.

Downregulation of *SQS* impacted concentrations of isoprenoid pathway intermediates in a tissue-specific manner. In stem tissues, MVA pathway intermediates acetyl co-A and mevalonate showed significantly reduced concentrations in *SQSi* lines compared with controls (Figure 5, Table S1). In contrast, for root tissues, the mevalonate concentration was higher in the *SQSi* lines and both hydroxymethylglutaryl coenzyme A (HMG-CoA) and GPP were enriched vs. WT (but not VC). Mevalonate was also slightly enriched in leaf but not to statistical significance.

Entering the MEP pathway, pyruvate was detected at the highest levels of all upstream isoprenoid pathway compounds in all tissues and all genotypes, as might be expected for a primary metabolite [9] (Table S1). Pyruvate concentrations for *SQSi* lines did not vary significantly from controls (except higher pyruvate in stem for SQSi-M). However, the important intermediate 2-C-methylerythritol 4-phosphate (MEP) was higher in stem and root for *SQSi* lines compared to WT (Figure 5, Table S1). In addition, HMBPP was present at higher concentrations in root tissue of *SQSi* lines compared to controls. In contrast, MEcPP (2-C-methyl-d-erythritol 2,4-cyclodiphosphate) was strongly and significantly reduced in root tissue of *SQSi* lines compared to the WT control (but not compared to the EV). Finally, CDP-ME (4-diphosphocytidyl-2-C-methylerythritol) levels were slightly enhanced in the leaf tissues of *SQSi* lines but not significantly (Figure 5, Table S1).

Other metabolites, including the prenyl phosphates (except GPP), were unchanged, not detected, or varied inconsistently in response to *SQS* downregulation (Table S1).

**Figure 6:**
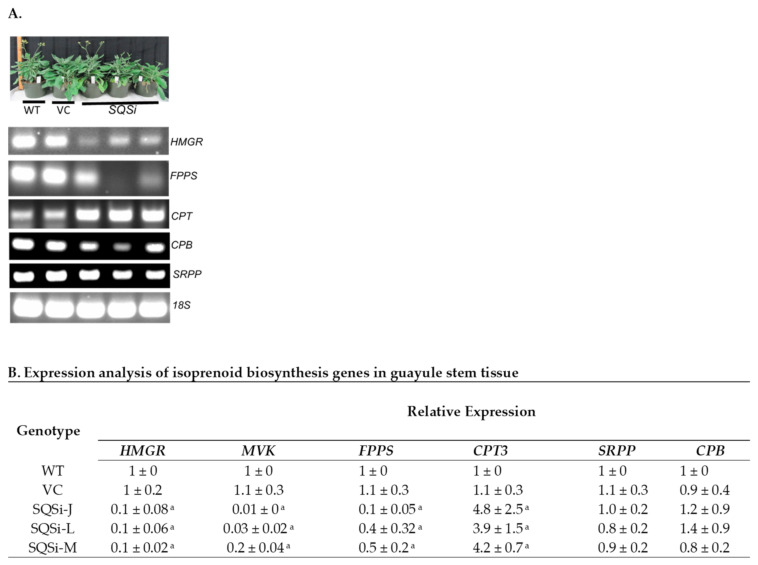
(**A**) Relative expression of metabolic and putative NR biosynthesis and structural genes in *SQSi* lines’ background. Plant phenotypes for controls and SQS lines, RT-PCR for the isoprenoid pathway, and natural rubber biosynthesis-associated genes. (**B**) Quantitative expression of isoprenoid biosynthesis genes. Expression levels in stem tissue were compared to WT and normalized to the 18S reference gene. Values are averages of three biological replicates +/− SD; (^a^) indicates a significant difference in comparison to *WT* at *p* > 0.05. *HMGR* = 3-hydroxy-3methylglutaryl-coenzyme A reductase; *FPPS* = farnesyl pyrophosphate synthase; *SQS* = squalene synthase; *CPT* = *cis*-prenyltransferase; *SRPP* = small rubber particle protein; *CBP* = *CPT* binding protein.

Finally, the relative expression of isoprenoid metabolic and rubber biosynthetic pathway genes was determined by Q-RT-PCR. MVA pathway genes encoding *HMGR*, *MVK*, and *FPPS* were all consistently downregulated in *SQSi* lines compared to the WT and VC control plants (Figure 6). Putative rubber biosynthetic genes encoding *SRPP* and *CBP* showed no difference for *SQSi* lines, but, remarkably, *CPT3*, the rubber-particle associated *cis*-prenyl transferase, was significantly upregulated in *SQSi* lines.

## 3. Discussion

Plants synthesize and accumulate an immense variety of isoprenoid metabolites under complex developmental and environmental controls. Strategically, metabolic engineering of a key enzyme in a crucial biosynthetic pathway can be a powerful approach to increasing a valuable end product. Thus, the hypothesis was posited that by diverting the carbon metabolic flux away from squalene synthesis, the production of NR would be increased in guayule.

Dong, Ponciano et al. [35] reported a single copy *SQS* gene in guayule, moderately expressed in stem tissue of field plants, which was the target of our RNAi construct (Figure 1A). Constitutive downregulation of *SQS* using this construct was successful based on gene expression results (Figure 1C) and the confirmation of lower squalene concentrations in stem tissue compared with controls (Figure 2A). We showed that squalene was more concentrated in stem tissues (Figure 2A), where, frequently, most rubber biosynthesis occurs, supporting the notion that NR initiation and squalene synthesis compete for the same substrate (FPP) in the same tissue.

The extent of reduction of squalene (~60%) was quite similar to that reported in other plants, *A. annua* [27] and *W. somnifera* [28]. Despite lowered squalene levels, in greenhouse/growth chamber *SQSi* guayule plants, pools of substrate, i.e., FPP, were not detected. Since FPP is a precursor to dozens of downstream products, it may be too quickly metabolized for detection. However, when SQS was downregulated, GPP levels in root tissue were enhanced, perhaps due to the downregulation of *FPPS*. Results then suggest available FPP and/or GPP (as suggested by the positive correlation between GPP and rubber (*p* < 0.05, Figure S3)) contributed to increased natural rubber production in root tissue.

In *Taraxacum kok-saghyz*, another rubber-producing plant, squalene content in rubber-producing root tissues was not affected by the downregulation of *SQS* [36]. However, downregulation of *SQE*, which catalyzes the next step in the metabolic pathway, increased squalene content by 27-fold, with no effect on isoprenoid biosynthesis (pentacyclic triterpenes and sterols; NR not reported).

Isoprenoid pathway regulation is known to be complex. Silencing of *SQS* in *W. somnifera* resulted in the upregulation of 3-hydroxy-3-methylglutaryl coenzyme A reductase (*HMGR*) and other MVA biosynthetic genes [28], attributed to a feedback regulation mechanism. In tobacco (*Nicotiana tabacum*), inhibition of *SQS* likewise upregulated *HMGR* [37]. In contrast, we found that downregulation of *SQS* in guayule lowered the expression of *HMGR, FPPS,* and *MVK* (Figure 6). High levels of FPP are known to downregulate the expression of *HMGR* in humans [38,39] and insects [40], and prenyl phosphates similarly inhibit *MVK* in animals [41,42]. In addition, it is well recognized that excess prenyl phosphates are inhibitory in the microbial production of terpenes [43,44].

Less is known about MVA feedback regulation by prenyl phosphates in plants. However, Hepper and Audley [45] identified HMGR activity as rate-controlling in Hevea rubber biosynthesis, and its mevalonate kinase activity was inhibited by GPP, FPP, and GGPP [46]. In our study, the MVA pathway was clearly impacted; when *HMGR*, *MVK*, and *FPPS* were downregulated in *SQSi* lines, higher concentrations of their respective substrates (HMG-coA, mevalonate, and GPP) accumulated, mainly in root tissues (Figure 5; Table S1). Taken together, the results suggest the intriguing possibility that GPP may be the preferred rubber biosynthesis initiator [47] in guayule root tissue and/or in the absence of FPP. Further evidence indicates that elevated GPP was responsible for the observed negative feedback regulation in the MVA pathway in guayule or that the concentration of FPP needed to suppress gene transcription was below detection limits (0.01 μg/g dw tissue).

Interestingly, in stem tissue, expression of the gene encoding rubber biosynthesis-associated *cis*-prenyl transferase 3 (CPT3) significantly increased in *SQSi* lines compared to WT controls, while levels of the gene encoding CPT3-binding protein (CBP) were unaffected (Figure 6), indicating these genes are regulated differently. CPT3 and CBP proteins are hypothesized to form an enzymatic complex (along with other yet-to-be-identified factors) necessary for rubber biosynthesis [48,49]. Despite the fact that *CPT3* transcription is stimulated by MVA pathway disruptions, no increase in rubber was detected in stem tissue, possibly due to the unchanged levels of *CBP* expression and concomitant translation to form the necessary active enzymatic complex. Expression levels of the gene encoding SRPP, a protein known to associate with the rubber particle, were also unaffected in stem tissue of *SQSi* lines compared to controls. SRPP has an indirect rubber biosynthesis function [50,51], and it appears to be insensitive to MVA pathway changes.

Characterization of isoprenoid pathway intermediates in this study revealed new insights beyond phenotypes for engineered guayule. In WT plants, for example, higher concentrations of HMBPP, IPP/DMAPP, and FPP found in the leaf are consistent with reported high terpene resin content in guayule leaves [52,53,54]. Considering the MEV pathway as a whole, tissue-specific accumulation patterns of mevalonate were, intriguingly, the inverse of the accumulation of rubber (Figure 4). Root tissues showed less accumulation of mevalonate compared to stems and lower IPP/DMAPP compared to leaves, suggesting higher flux through the MVA pathway, which may explain why more rubber was concentrated in root tissues for these greenhouse plants. Interestingly, the accumulation of MEcPP in the stem and root, as well as HMBPP in the leaf, suggests that further optimization of the MEP pathway (e.g., overexpressing *ispG* and *ispH*) could result in greater production of rubber in these tissues.

While the MVA pathway is considered the preferential route for C_30_ isoprenoid formation in plants [55], downregulation of *SQS* in guayule impacted the metabolite pools in both the MVA pathway in the cytosol and the MEP pathway in chloroplasts. Evidence for active exchange between intermediates in these pathways has been established [56,57,58] but poorly understood in guayule. In our investigation, up to five-fold higher mevalonate concentration and up to double MEP concentrations were found in plant tissues of *SQSi* guayule. While the feedback mechanisms controlling both the MVA and MEP pathways are numerous and complex [55], our results provide evidence of crosstalk in the feedback regulation of both isoprenoid pathways in guayule.

The metabolite data also suggests that rerouting carbon from squalene, especially in the stem, might increase rubber production, consistent with our strategy. In our work, downregulation of squalene synthase reduced squalene content by more than half in guayule stems, potentially creating a pool of excess FPP. If half of the excess FPP had been used to initiate new rubber molecules, about 33,000 μg/g dw rubber would have resulted, an enormous increase in rubber content of +3% dw, which was not observed. This strongly indicates that IPP, not detected in stem tissues, is the limiting factor under the conditions of our study. While the quantification of upstream isoprenoids in plant tissues is challenging and infrequently reported, metabolomics information holds promise to further reveal new insights, guiding future strategies in natural rubber bioengineering.

In summary, downregulation of squalene synthase in guayule successfully increased natural rubber production, but not to the extent of squalene reduction on a carbon count basis. Isoprenoid pathways producing NR and squalene substrates were perturbed in quite unpredictable ways. Importantly, analysis of MVA and MEP pathway intermediates has provided the first evidence of cellular crosstalk between these essential metabolic pathways in guayule.

## 4. Materials and Methods

### 4.1. Plant Transformation Plasmid Construction

Plasmid *pPZP200* [59] provided the backbone for generating the pND9 and pND9_*SQSi* constructs used in this study. Plasmid pND9 consisted of a shortened potato polyubiquitin promoter (409-Ps) [60], driving the neomycin phosphotransferase II (*nptII*) gene for conferring kanamycin resistance [61], and an octopine synthase terminator (Ocs-T) [62], along with a MtHP-P promoter [63] controlling β–glucuronidase plus (*GUSPlus*) gene [64] expression, and a ubiquitin 3 terminator (Ub3-T) [65]. The plasmid pND9_*SQSi* was constructed by replacing the *GUSPlus* gene with an inverted repeat of the partial guayule *squalene synthase* gene (*SQS*) (see below and Figure 1).

The SQS gene sequence was obtained from the published sequence in the NCBI database (GenBank accession no. HQ131832.1). This sequence was used to blast the Guayule Genomic Resources (https://probes.pw.usda.gov/Guayule/, accessed on 25 March 2022) to obtain the guayule SQS sequence (clc8thAssembly_contig_273481). Forward primer 5′-AAAACAAATATCATCCGTGATTATCTAGA-3′ and reverse primer 5′-CTGAGGAATAGCACAAAACCTGA-3′ were used to PCR the guayule cDNA template. The resulting 252 bp PCR product was subcloned into a pGEM-T vector (Promega, Madison, WI, USA) and sequenced to confirm its integrity. Subsequently, an inverted repeat was made by joining an NcoI-BglII reverse fragment and a 552 bp BamHI-SpeI BAR gene [66] and SpeI-SacI forward fragment together, replacing the GUSplus gene in pND9. The plasmids pND9 and pND9_*SQSi* were used to transform *Agrobacterium* EHA101 [67] competent cells. The transformed *Agrobacterium* EHA101, harboring either pND9 or pND9_*SQSi,* were used to transform guayule G7-11, a publicly available USDA germplasm line, distributed as AZ-2 [68].

Plasmid *pND9*-*SQS* (Figure 1A) was generated by replacing the *GUS* gene with a full-length *SQS* gene (GeneBank accession no. sT7aPBC069F0650.ab1). Plasmid *pND9-SQSi* was constructed by replacing the GUS gene with an inverted repeat (Figure 1A). The repeat contained a reverse complimentary 252 bp in its 5′ end and the forward 252 bp in its 3′ end. A BAR gene [66] of 552 bp was inserted into the middle of this inverted repeat. The plasmids *pND9* and pND9-*SQSi* were used to transform *Agrobacterium EHA101* competent cells [67]. The transformed *Agrobacterium EHA101,* either harboring *pND9* or *pND9*-*SQSi*, were used to transform into guayule *G7*-*11*. Wildtype and transgenic guayule lines were maintained in tissue culture, as previously described [12].

### 4.2. Agrobacterium and Leaf Tissue Transformation

*Agrobacterium* and leaf tissue transformation were performed based on Dong et al. methods [12,69]. For *Agrobacterium* transformation, the overnight *Agrobacterium* culture was prepared by inoculating 20 µL glycerol stock into a 50 mL Falcon tube containing 5 mL Luria-Bertani (LB) medium plus 40 mg/L rifampicin and 200 mg/L streptomycin, shaking vigorously at 200 rpm at 28 °C for 16 h. The suspension was then centrifuged for 15 min at 3500 rpm at room temperature. The supernatant was discarded, and the pellet was re-suspended in 25 mL of inoculation solution [1/10 Murashige and Skoog (MS) salts [70] plus 6-benzylamnopurine (BA; 2 mg/L), 1-naphthaleneacetic acid (NAA; 0.5 mg/L), glucose (10 g), acetosyringone (200 µM), pluronic F-68 (0.05%), and pH 5.2].

For leaf transformation, leaves were cut from plants grown in 1/2MSI0.1 [1/2MS containing indole-3-butyric acid (IBA 0.1 mg/L), sucrose 15 g/L, agar 8 g/L, pH 5.8] in Magenta boxes]. The adaxial side of each leaf was placed facing up in a Petri dish containing 5 mL *Agrobacterium* suspension. The leaf was cut into ~10 mm strips and immediately placed in an empty Petri dish in a non-overlapping manner. When this Petri dish was full, all leaf strips were blotted with the filter paper and placed into another empty Petri dish. The Petri dish was sealed by parafilm and placed in the dark. After 3 days, the leaf strips were transferred to MSB1T400 [MS medium with BA 1 mg/L, NAA 0.25 mg/L, sucrose 30 g/L, agar 8 g/L, and timentin 400 mg/L] for recovery under low light. After 5 days, the leaf strips were then transferred to MSB0.75TK30 [MS medium containing BA 0.75 mg/L, NAA 0.25 mg/L, sucrose 35 g/L, agar 8 g/L, timentin 250 mg/L, and Kanamycin 30 mg/L] for selection under low light. After two weeks, the leaf strips were subcultured to the same MSB0.75TK30 medium but grown under high light. Subculturing was performed every 2 weeks under high light until green shoots emerged. Green shoots 10 mm and longer were transferred to 1/2MSI0.1TK10 [same as 1/2MSI0.1 but with timentin (200 mg/L) and kanamycin (10 mg/L)] for root development. After 1–2 months, shoot tips of the rooted plantlets were transferred to 1/2MSI0.1TK10 for micropropagation or transplanted into soil.

### 4.3. Genomic DNA and RNA Extractions and PCR and qPCR Analyses

Plant DNA was extracted using a Sigma GenElute Plant Genomic DNA Miniprep Kit (Sigma-Aldrich, Carlsbad, CA, USA). Approximately 150 mg leaf tissue was cut from plants grown in tissue-culture, placed into 2 mL tubes, and snap-frozen in liquid nitrogen. A bead was added to pulverize the tissues into a fine powder at a frequency of 30/s for 1 min using the mixer mill MM 400 tissue lyser (Verder Scientific, Inc., Newtown, PA, USA). PCR was carried out in 50 µL of a mixture containing GoTaq Green Master Mix (Promega Corp., Madison, WI, USA), 200 ng guayule genomic DNA or 20 pg plasmid DNA, and 1 µM of *BAR*-specific primers:

5′-ATGAGCCCAGAACGACGCCCGGCC-3′ and 5′-GATCTCGGTGACGGGCAGGACCGG-3′. After heating the samples to 95 °C for 2 min, the reaction proceeded with 40 cycles of 95 °C for 30 s, 71 °C to amplify the product ~0.5 kb in the *SQSi* lines for 30 s, and 72 °C for 1 min. A final elongation step was carried out at 72 °C for 5 min. PCR products were separated by electrophoresis on a 1% (*w*/*v*) agarose gel.

The iScript cDNA synthesis kit (Bio-Rad, Hercules, CA, USA) was used to synthesize complementary DNA (cDNA) for both RT-PCR gel electrophoresis analysis and quantitative RT-PCR, following manufacture instructions. RNA extraction, cDNA synthesis, and qPCR analysis were performed as previously described [71], with the following changes: for qPCR analysis, cDNAs were diluted 1:10 in 15 µL volume reactions containing iQ SYBR Green Supermix (Bio-Rad, Hercules, CA, USA). Thermocycler 7500 Fast Real-Time PCR system (Applied Biosystem, Foster City, CA, USA) conditions: 95 °C pre-incubation for 3 min; amplification for 40 cycles at 95 °C for 15 s and 60 °C for 30 s; the dissociation stage for 95 °C for 15 s, 60 °C for 1 min, and 95 °C for 15 s. Each qPCR run was performed with three independent tissue samples, each sample having two technical replicates. The *18S* gene was used as an internal control with the following respective forward and reverse primer sequences: 5′-CAACAAACCCCGACTTCTGG-3′/5′-CACCCGTCACCACCATAGTA-3′. Target genes forward/reverse primer pair sequences are: *SQS*, 5′-GATCCCAATGCCACAACTACAA-3′/5′-CCAGAGGCCTTGCATATTTTCT-3′; *HMGR*, 5′-ATGGGCATTTCTGGAAACTATTG-3′/5′-CTTCCCTCGCCCTTCTATCC-3′; *MVK*, 5′-GGCATCCTGATGCTATGACA-3′/5′-GCATTGGAGCAAACCTTGAT-3′; *FPPS*, 5′-TCAACGATCCTGCCTTCGA-3′/5′-TCCAGGTACGTTGTAGTCAAGCA-3′; *CPT3*, 5′-GCTTCTTTTTCGGGTCATTTCA-3′/5′-TGCCAAGAATCCGGCTTTAT-3′; *CBP*, 5′-GGCGGTCATCATGGAGAGA-3′/5′-GATTGGCTACTGCACTATCATTGG-3′; and *SRPP*, 5′-GTGGCCAACACATTGTACGTAAA-3′/5′-TTCTCAGCTACCGGCTCGTAGT-3′.

### 4.4. Rubber and Resin Extractions

The top part (stems and leaves) of approximately two-month-old tissue culture plantlets or tissues separated from 5-month-old greenhouse/growth chamber plants were separated from the medium/soil and lyophilized for 48 h. The dried tissues were placed in a 50 mL stainless steel grinding jar containing a grinding ball, frozen in liquid nitrogen for 5 min, and finely ground using the mixer mill MM 400 at a frequency of 30/s for 1 min. Three hundred milligrams (0.3 g) of pulverized tissues were loaded into 11 mL stainless steel-sand containing extraction cells (Dionex, Sunnyvale, CA, USA). Three sequential extractions were performed: 1. acetone, to remove resinous material and the low molecular weight organic solubles; 2. methanol, to remove chlorophyll and other alcohol-soluble materials; 3. cyclohexane, to remove rubber. Natural rubber was quantified gravimetrically (from ASE vials). The percent (%) rubber is the amount (% dw) of cyclohexane extract from 0.3 g dried tissue.

### 4.5. Transplanting and Plant Architecture Measurements

Plantlets from tissue culture were carefully uprooted from the ½ MS medium and transplanted into a pot (6″ diameter × 4.25″ depth; ITML Horticulture, Canby, OR, USA) with Sunshine Mix potting soil (Sungro Horticulture, CA, USA). Plants were grown in the greenhouse for 1 month and later transferred into the growth chamber at 40–45% relative humidity, 25 °C under cool-white fluorescent light (50 µmol m^−2^ s^−1^ 16/8-h day/night photoperiod). The design included 3 replications per line, placed randomly in a complete block design. There was one G7-11, one pND9 empty vector control, and three *SQSi* independent lines. Plants were watered and fertilized as needed. SPAD measurements were made prior to harvest, reported as an average of 3 leaves. Plant biomass, height, width, number of stems, flowering buds, stembark thickness were measured on five-month-old plants. Plants were photographed prior to harvest.

### 4.6. Squalene Extraction and Purification

Fresh leaf, stem, and root tissues from 5-month-old growth chamber grown plants were harvested, dried, and ground as described above. Approximately 300 mg of ground tissue was placed into an 11 mL ASE extraction cell (Dionex, Sunnyvale, CA, USA) with Ottawa sand as a dispersant (Fisher Scientific, Waltham, MA, USA). Crude resin containing the squalene was extracted with acetone at room temperature, then evaporator dried at 50 °C with 15 psi N_2_.

Column purification was used to separate the squalene-containing fraction from other components. A 1 × 20 cm activated aluminum column was carefully poured (Alumina 80–200 mesh chromatography grade; Spectrum Chemical MFG Corp) and prepared using hexane (certified ACS, Fisher Chemicals, Rockingham County, NH, USA) to remove all voids or bubbles. The column was eluted with 15 mL hexane. Plant tissue extracts, re-suspended in 2 mL fresh acetone (HPLC grade, Fisher Chemical, Fair Lawn, NJ, USA), were poured onto the column, then eluted with 35 mL hexane/acetone (70:30). The first 10 mL was discarded as void volume; then, successive 5 mL fractions were collected. Preliminary experiments showed squalene eluted in the first fraction. For experimental extracts, the first 10mL fraction was collected to assure quantitative recovery. Fresh new columns were prepared for all 72 samples so that there was absolutely no chance of contamination within or between tissue types. Root sample fractions were water-clear; for stem and leaf tissues, squalene was co-extracted with yellow/green carotenoids. The purified extracts were evaporated using a TurboVap LV (Biotage, Charlotte, NC, USA) evaporator at 40 °C with 15 psi N_2_. The purified, dried material was then re-suspended in 1 mL fresh acetone.

### 4.7. Squalene Quantification by GC-MS

The separation and quantification of squalene were conducted on a DB-5ms capillary column (30 m length, 250 µm internal diameter, and 0.25 µm film thickness: Agilent Technologies J&W Scientific, Santa Clara, CA, USA, part number 122-5532G) using an Agilent Technologies 6890 Series gas chromatograph (GC) system. The GC system was coupled to a Leap Technologies COMBIPAL (CTC Analytics, Zwingen, Switzerland) autosampler. A sample injection volume of 1 µL was used throughout, and the sample was injected in the splitless mode. Helium was used as the carrier gas at a constant flow rate of 1.3 mL/min. For the GC temperature gradient program, initial isothermal heating of 210 °C was applied for 1 min; the temperature was then increased to 280 °C at a rate of 20 °C/min for 3.5 min, and held at 280 °C for 3 min, increased to 300 °C at a rate of 20 °C/min for 1 min, and further held at 300 °C for 3 min. The total fun time was 11.5 min. The Agilent Technologies 6890 Series GC system was coupled to an Agilent 5973 network mass selective detector. The temperature of the inlet, transfer interface, and ion source was set to 250, 250, and 230 °C, respectively. A solvent delay of 2 min was implemented throughout the course of the experiment. Electron impact ionization (70 eV) was used, and an acquisition rate of 0.98 cycles/s was employed. The fragment ions used for selected ion monitoring experiments were 69, 81, 136, 137, and 410 *m*/*z* (the parent ion). The identification of squalene was based on the retention time and the abundance of the fragment ions. The fragment ion at 69 *m*/*z* was used for quantification since it was the most abundant ion in the mass spectrum of squalene. Data acquisition and analysis were performed by the Chemstation software package (from Agilent Technologies). Squalene from the guayule extract was quantified via a seven-point calibration curve, ranging from 1.5625 to 100 µM. The R^2^ coefficients for the calibration curves used in this study were >0.99 (with a 0-intercept setting).

### 4.8. Quantification of Isoprenoid Intermediates by LC-MS

LC-MS was used to analyze metabolite profiles according to a previous method whose LC [72] and MS [73] conditions have been, respectively, previously described.

### 4.9. Statistical Analysis

Quantitative variables are expressed as the arithmetic means ± standard deviation (SD) or standard error (SE) as specified. A two-tailed paired Student *t*-test was used to measure the significance of difference between transgenic lines and wildtype, as presented by *p*-value. Multivariate analysis was performed to explore the relationships of precursor and intermediate compounds involved in natural rubber and squalene biosynthesis across all control and transgenic lines with different tissue. Measurements were pre-treated by auto-scaling. Pearson’s correlation-based hierarchical cluster analysis was used to group variants by metabolites and individual plant lines (tissue and genotype), respectively. Pearson’s correlation analysis was also used to explore univariate associations between each pair of individual studied metabolites. Correlation coefficients with *p*-values < 0.05 were considered significant.

## Data Availability

All data presented in this study is available in the article and supplementary material. In addition, the analysis code that supports the findings of this study is available at GitHub https://github.com/chenchen-dong/Squalene_Plots (accessed on 28 March 2022).

## Supplementary Materials

The following supporting information can be downloaded at: https://www.mdpi.com/article/10.3390/metabo12040303/s1, Figure S1: Isoprenoid pathway schematic with squalene synthase downregulation; Figure S2: Growth phenotypes of WT guayule and SQS transgenic lines; Figure S3: Correlation matrix heatmap of precursor and intermediate compounds involved in natural rubber and squalene biosynthesis; Table S1: Metabolites content (µg/g) of 5-month-old guayule tissues.
