# Supplementary material for: Downregulation of Squalene Synthase Broadly Impacts Isoprenoid Biosynthesis in Guayule
Source: Metabolites. 2022 Mar 29;12(4):303. doi: 10.3390/metabo12040303 (PMC9030042; doi:10.3390/metabo12040303)
Supplement: Supplementary file 1 [file metabolites-12-00303-s001.zip › metabolites-1644847-supplementary.pdf]

Article

Downregulation of squalene synthase broadly impacts isoprenoid biosynthesis in guayule

Dante Placido[1], Niu Dong[1], Basha Amer[2], Chen Dong[1], Grisel Ponciano[1], Talwinder Kahlon[1], Maureen Whalen[1], Edward E. K. Baidoo[2] and Colleen McMahan[1,*]

Supplementary Materials:

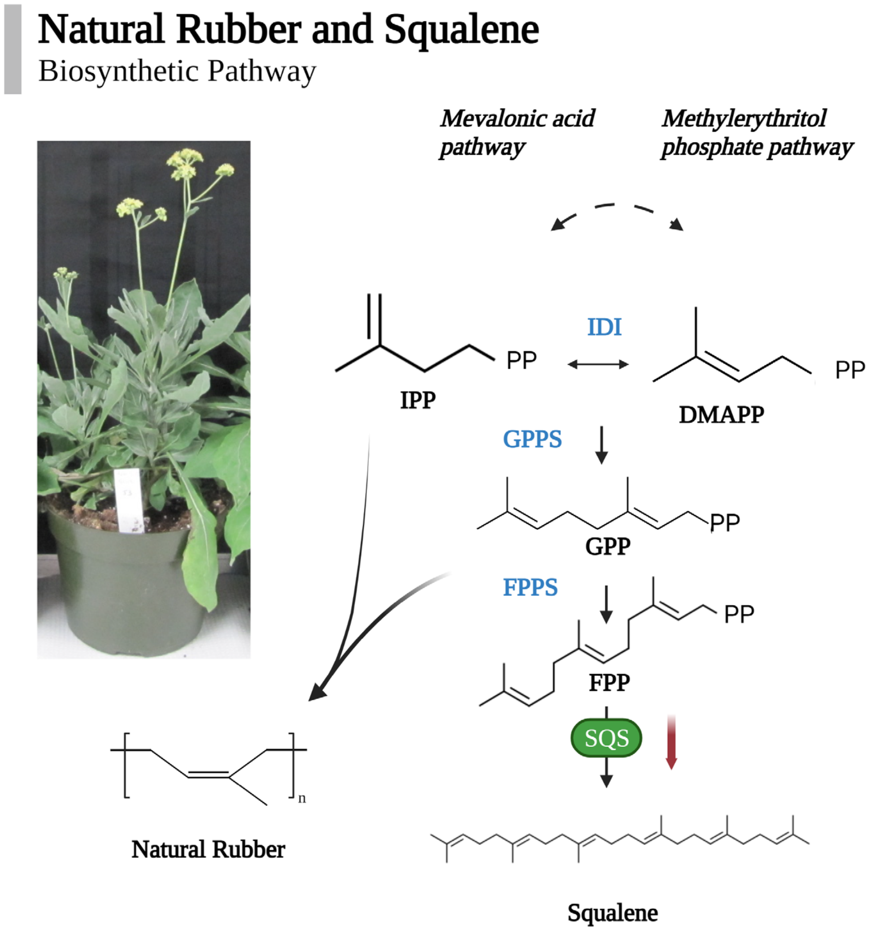

Supplementary Figure S1: Isoprenoid pathway schematic with squalene synthase downregulation: 1) the cytosolic Mevalonic acid pathway produces the 5 carbon isopenentyl pyrophosphate (IPP). The Methylerithritol phosphate pathway (chloroplast) produces dimethylallyl diphosphate (DMAPP), which can be isomerized by isopentenyl isomerase (IDI) to IPP. Other key components include: geranyl diphosphate (GPP) and its synthase (GPPS), farnesyl diphosphate (FPP) and its synthase (FPPS), squalene and natural rubber. Downregulation of squalene synthase (SQS) by RNAi technology is represented by the red arrow.

A.

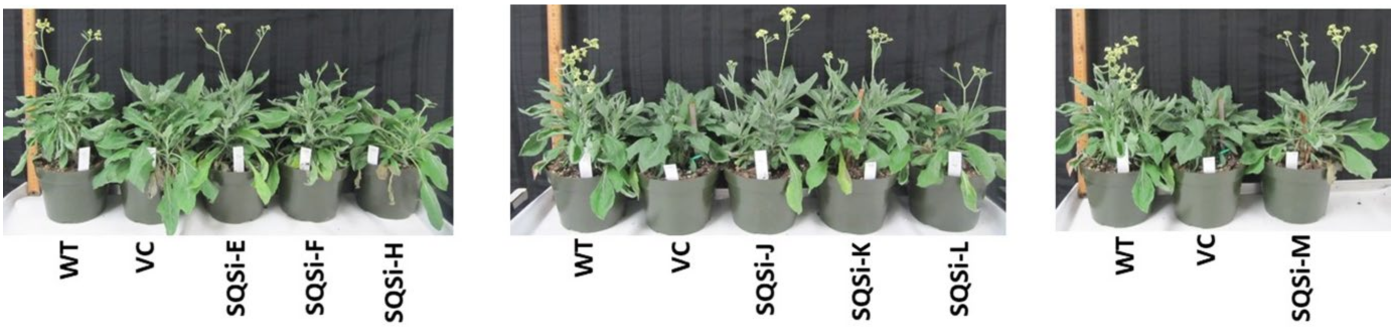

B.

**Genotype**	**Plant phenotype characteristics**						
	**Height (cm)**	**Width (cm)**	**Stembark thickness (mm)**	**Number of branches**	**Number of flowering buds**	SPAD reading	Dry Weight (g)
WT	22 ± 2	30 ± 3	2.1 ± 0.4	8 ± 1	59 ± 8	49 ± 3	11 ± 1
VC	22 ± 3	35 ± 4	1.7 ± 0.1	5 ± 1 [a]	5 ± 4 [a]	51 ± 1	13 ± 1
SQSi-J	25 ± 2	30 ± 2	1.6 ± 0.2	8 ± 2	50 ± 7	57 ± 4	12 ± 1
SQSi-L	23 ± 2	30 ± 3	1.9 ± 0.4	7 ± 1	33 ± 19	47 ± 1	11 ± 2
SQSi-M	23 ± 3	28 ± 2	1.3 ± 0.1 [a]	6 ± 1	17 ± 6 [a]	53 ± 6	9 ± 1

Supplementary Figure S2. Growth phenotypes of WT guayule and SQS transgenic lines. **A.** Images of 5-month-old plants, **B.** Phenotype characteristics: all values average of 3 biological replicates, +/- one standard deviation. 'a' indicates significantly different than WT control per student T-test at p>0.05.

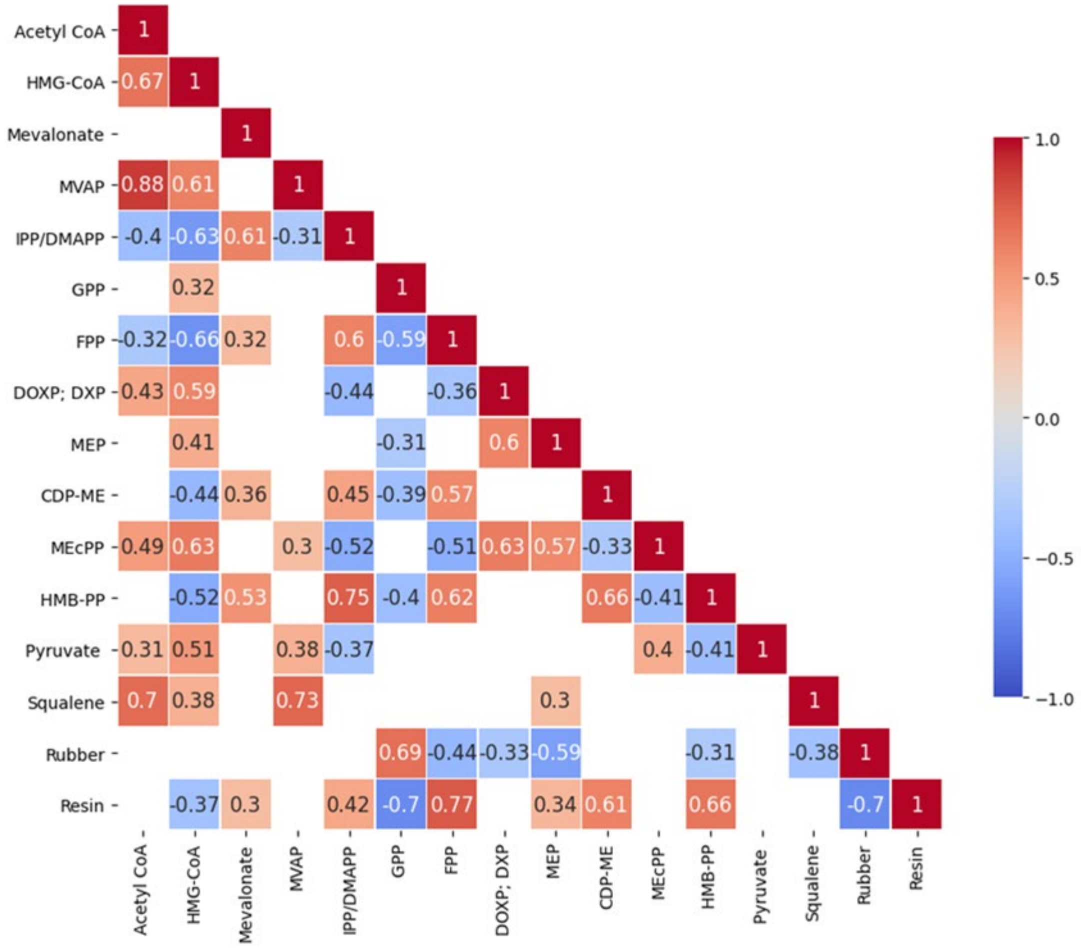

Supplementary Figure S3. Correlation matrix heatmap of precursor and intermediate compounds involved in natural rubber and squalene biosynthesis across all control lines and SQSi lines, with leaf, stem and root tissue. Negative correlations in blue, positive in red. Color intensity are proportional to the correlation coefficients, which are the numbers displayed for each pair. Correlations with p-value > 0.05 are considered as insignificant and did not show in the matrix.
